# Bone and joint quality remains impaired in long-term controlled acromegaly: an HR-pQCT study

**DOI:** 10.1210/jendso/bvag135

**Published:** 2026-06-22

**Authors:** Christian Rosendal, Frederik Østergaard Klit, Annika Vestergaard Kvist, Peter Vestergaard, Jakob Dal

**Affiliations:** Department of Endocrinology, Aalborg University Hospital, Gistrup 9260, Denmark; Department of Endocrinology, Aalborg University Hospital, Gistrup 9260, Denmark; Steno Diabetes Center North Denmark, Aalborg University Hospital, Gistrup 9260, Denmark; Steno Diabetes Center North Denmark, Aalborg University Hospital, Gistrup 9260, Denmark; Steno Diabetes Center North Denmark, Aalborg University Hospital, Gistrup 9260, Denmark; Department of Endocrinology, Aalborg University Hospital, Gistrup 9260, Denmark; Steno Diabetes Center North Denmark, Aalborg University Hospital, Gistrup 9260, Denmark

**Keywords:** acromegaly, pituitary disease, arthropathy, bone quality, vertebral fractures

## Abstract

**Context:**

Joint and bone disorders are frequent, disabling complications of acromegaly that progress despite biochemical remission. HR-pQCT provides a precise assessment of bone and joint quality.

**Objective:**

To evaluate joint and bone quality in patients with long-term controlled acromegaly.

**Design:**

Cross-sectional study.

**Participants:**

17 acromegaly patients and 17 age- and sex-matched controls.

**Measures:**

HR-pQCT of hand joints, tibia, and radius; bone microindentation; vertebral fracture assessment; dual X-ray absorptiometry; circulating bone biomarkers; questionnaires.

**Results:**

Patients with acromegaly (mean age: 61.8 years, mean disease duration: 13.1 years, mean IGF-I xULN: 0.9) had wider joint spaces of the second (2.85 vs 2.68 mm, *P* = .004) and third (2.92 vs 2.80 mm, *P* = .037) metacarpophalangeal joints, the latter with a higher risk of osteophytes (50% vs 11.8%, *P* = .017). Vertebral deformities were more common (88% vs 47%, *P* = .01), more numerous (2.1 vs 0.7, *P* < .001), and associated with higher spinal deformity indices (2.6 vs 1.3, *P* = .04) in participants with acromegaly. HR-pQCT demonstrated lower trabecular bone mineral density in the radius (114.8 vs 150.3 mg/cm^3^, *P* = .008) and tibia (129.3 vs 166.2 mg/cm^3^, *P* = .002), as well as fewer (1.63 vs 2.11 mm^−1^, *P* = .001) and thinner (0.056 vs 0.064 mm, *P* = .005) trabeculae. Bone and joint abnormalities increased with age, and the spinal deformity index correlated positively with arthropathy indices, only in the acromegaly group.

**Conclusion:**

Long-term, biochemically controlled acromegaly is associated with structural joint alterations and impaired bone microarchitecture, even years after diagnosis.

Acromegaly is a rare endocrine condition [1, 2] characterized by hypersecretion of growth hormone (GH) from a benign pituitary adenoma [3], leading to several severe complications [4], both distressing for patients [5] and costly for health care systems [6]. No complication, however, is more deleterious to patients' quality of life (QoL) than the musculoskeletal complications of acromegaly [7, 8], which may progress over time, irrespective of disease control [9, 10].

Due to the trophic effects of GH and insulin-like growth factor 1 (IGF-I) on chondrocytes [11], acromegaly leads to cartilage growth, which causes periarticular ligament laxity, joint instability, increased mechanical stress, cartilage ulceration, and destruction [12], ultimately leading to manifest osteoarthritis [10, 12-15]. Unlike primary osteoarthritis, however, acromegalic arthropathy is characterized by preserved or widened joint spaces with severe osteophytosis [12, 15-19]. High-resolution peripheral quantitative computed tomography (HR-pQCT) has been used extensively to assess joint pathology in rheumatological disorders, as it provides detailed images of peripheral periarticular structures and enables precise quantification of structural abnormalities such as erosions and osteophytes [20], but also joint space volume [21, 22]. While osteoarthritis with severe osteophytosis is a well-established hallmark of acromegaly [12, 13, 15-19], this joint pathology has thus far not been studied using HR-pQCT.

The effects of GH and IGF-I excess on the skeleton are manifold [11], with the net result of compromised trabecular structure and quality but preserved—or even increased—areal bone mineral density (aBMD) on dual X-ray absorptiometry (DXA) imaging [11, 23-32]. The compromised quality of the trabecular bone compartment leads to a higher risk of vertebral fractures [10, 11, 24, 27, 29, 33-35], whereas the data on appendicular skeletal fractures are more divergent, with both reduced [10, 36] and increased [37, 38] risk being reported. Bone turnover markers have been found to be increased in active acromegaly [27], whereas sclerostin—an inhibitor of bone formation secreted by osteocytes—has shown a more divergent pattern as relates to acromegaly activity, with both significantly higher and lower values being reported [33, 39, 40], and without a clear relation to the occurrence of vertebral fractures [33]. Since aBMD measurements correlate poorly with bone quality in patients with acromegaly [11, 27, 30-32], alternate means for its assessment have been explored, including trabecular bone score [26, 28, 41], quantitative computed tomography (QCT) [30], cone beam tomography [42], HR-pQCT [23, 31, 32] and bone impact microindentation [43]. HR-pQCT provides high-resolution images of peripheral skeletal structures, with its smaller voxel size of 82 μm and shorter acquisition times setting it apart from MRI and QCT imaging modalities, and HR-pQCT is considered the gold standard for imaging peripheral bone microarchitecture [20, 44].

Bone impact microindentation, a minimally invasive procedure using a hand-held stylus to assess cortical bone structural integrity [45], has shown promise as a supplement to standard-of-care bone evaluation, since it provides information on in vivo bone quality and mechanical properties at the tissue level [45] not otherwise attainable. No studies have yet examined how these modalities intercorrelate with aBMD in patients with long-term, well-controlled acromegaly.

We aimed to study joint features using HR-pQCT and perform an integrated assessment of bone quality using HR-pQCT, DXA, bone impact microindentation, and vertebral fracture assessment (VFA), in patients with long-term controlled acromegaly compared to matched healthy controls.

## Materials and Methods

The study was conducted as a cross-sectional study, and the main outcomes of interest were the HR-pQCT-derived joint parameters (joint space volume, joint space width, and osteophyte number and diameter), with bone impact microindentation, DXA parameters, and bone-related HR-pQCT parameters (trabecular and cortical indices, specifically trabecular vBMD, number, thickness and separation, and cortical vBMD, porosity, and cortical pore diameter) as secondary outcomes.

### Participants

Participants with acromegaly were recruited from the outpatient clinic of Aalborg University Hospital, North Denmark Region, with the following inclusion criteria: Verified acromegaly diagnosis, in biochemical remission at the time of the study; ability to provide informed consent; preserved ambulatory function; stable replacement therapy of any pituitary deficiency.

The exclusion criteria were: diagnosis of osteoporosis necessitating medical treatment as per local guidelines [46]; conditions associated with secondary osteoporosis (severe kidney or liver dysfunction, malabsorption, multiple myeloma, primary hyperparathyroidism); treatment with drugs that impair bone quality; diagnosis of joint diseases unrelated to acromegaly; active drug or alcohol abuse; pregnancy; known allergy/hypersensitivity to local anesthetics or disinfectant, or other physical factors that render the subject unable to participate. Although a risk factor for secondary osteoporosis [47], diabetes was not a criterion for exclusion due to its close association with acromegaly [4]. For each participant with acromegaly, one sex- and age-matched control was identified through a database of volunteer participants.

Participants' weight and height were measured, and a smoking and diabetes history was recorded. For the acromegaly patients, disease-specific data on hormonal levels and acromegaly treatment were obtained from medical records. Biochemical disease control was defined as having an IGF-I standard deviation score ≤ 2 at the latest measurement.

All participants signed a written informed consent form before taking part in the study. The study was conducted in accordance with the Helsinki declaration, approved by the local ethics committee (case no. N20220041) and prospectively registered with www.clinicaltrials.gov (ID: NCT05752825).

### Study procedures

#### HR-pQCT

**Figure 1:**
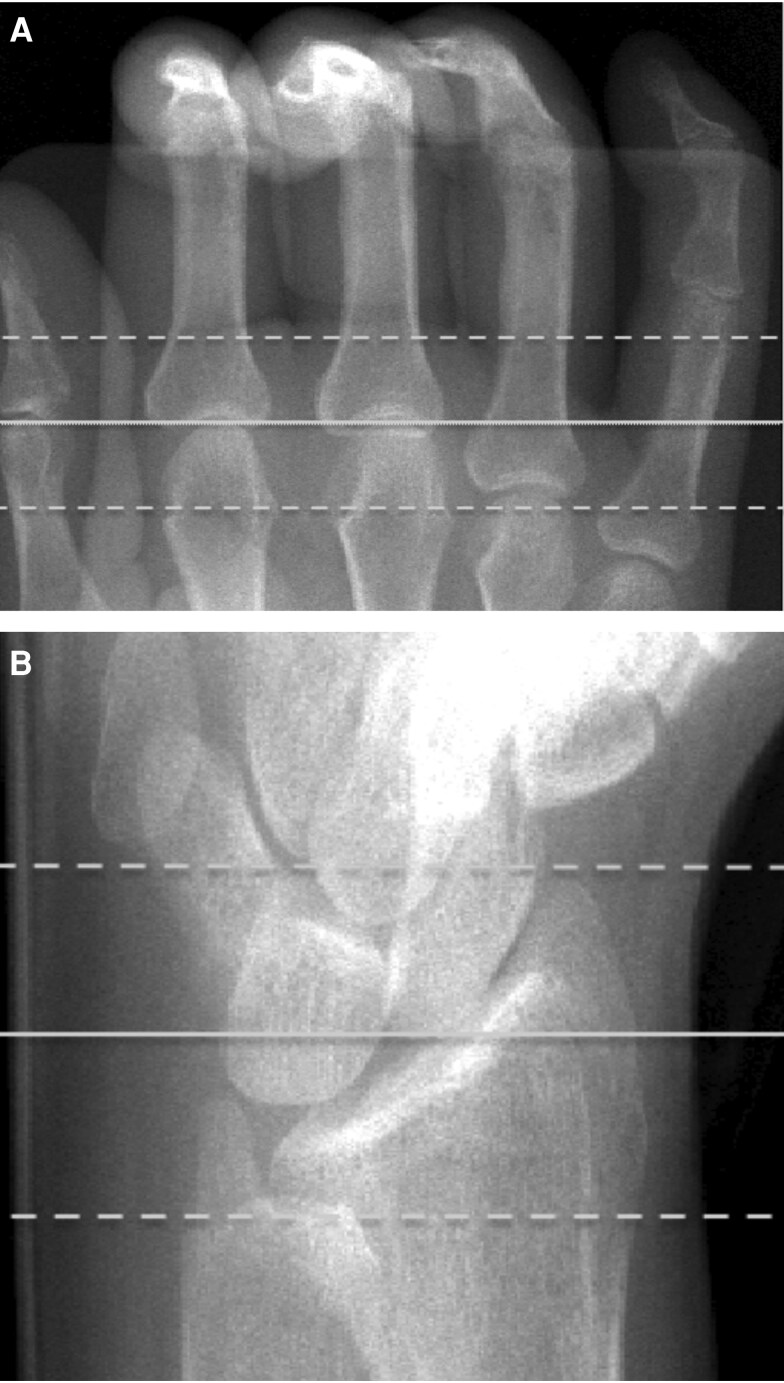
Placement of the reference lines for HR-pQCT joint imaging. A) MCP joints and B) Wrist joint.

The non-dominant distal radius and tibia were scanned with XtremeCT I (ScanCo Medical AG, Brüttisellen, Switzerland), as were the wrist joint and the second and third metacarpophalangeal (MCP) joints. For joint imaging, the reference line was placed at the midpoint of the radial articular surface curvature, and the concave articular surface at the base of the second proximal phalanx, respectively (Fig. 1). Two stacks of 110 slices each were recorded at each site, corresponding to 9.02 mm on each side of the reference line.

Based on the scans, bone and joint parameters were calculated using the software and scripts supplied by the manufacturer, based on a recent consensus statement [22].

The cortical bone compartments of the distal tibia and radius were analyzed for area (CtA), perimeter (CtPm), volumetric bone mineral density (CtvBMD), porosity (CtPo), pore diameter (CtPoDm), and thickness (CtTh), whereas the trabecular compartments were assessed for area (TbA), vBMD (TbvBMD), trabecular number (TbN), thickness (TbTh), separation (TbS), and bone volume/tissue volume ratio (BV/TV).

A daily quality control program was in place using a standardized phantom. The coefficients of variation (CV) were low (between 0.02% and 4.35%) [48].

**Figure 2:**
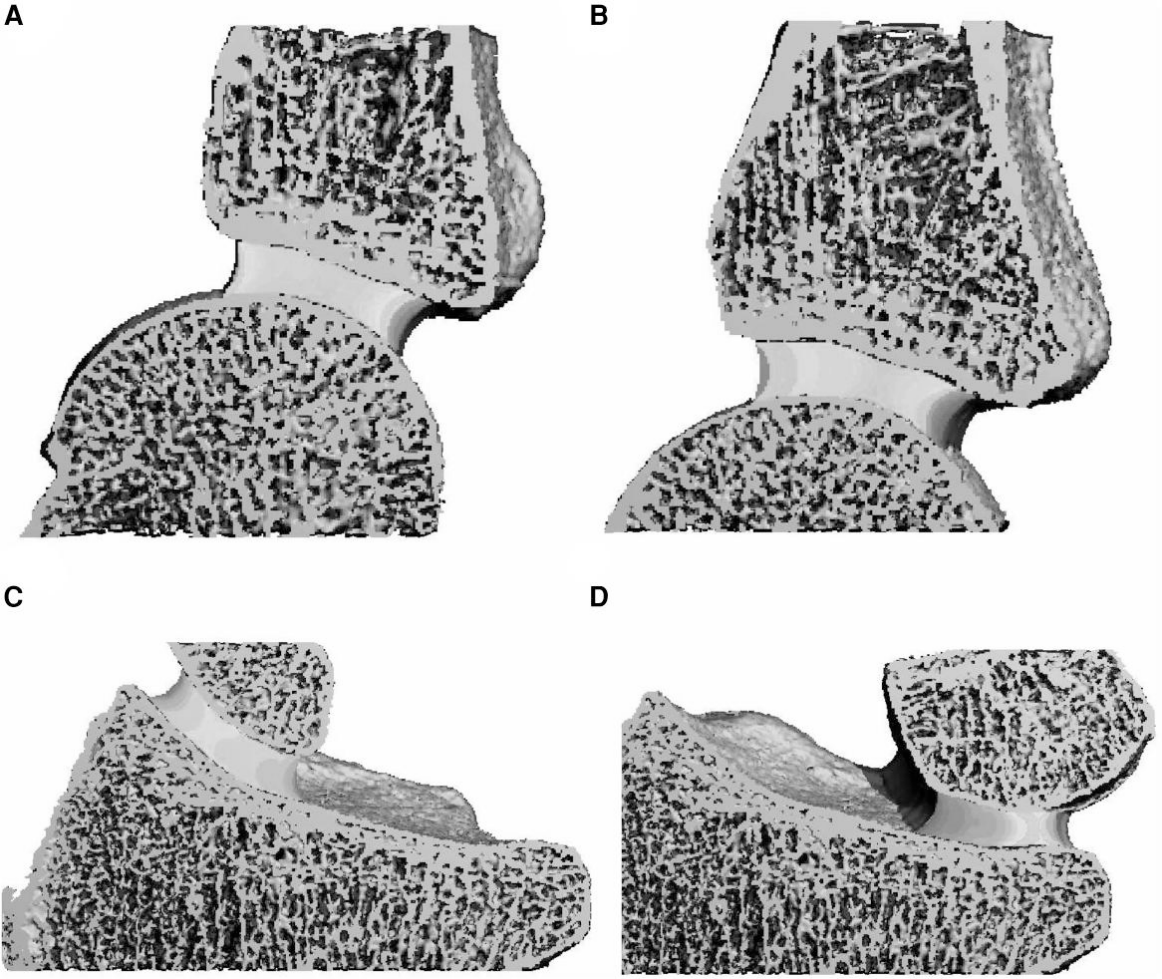
Examples of HR-pQCT joint space images. A) Second MCP joint, B) third MCP joint, C) radioscaphoid joint, and D) radiolunate joint.

For analysis of joint space, the wrist joint was divided into 2 separate entities, ie, the radioscaphoid and radiolunate joint, and the wrist and MCP joints were assessed for joint space width (JSW), volume (JSV), maximum and minimum joint space width (JSW_max_ and JSW_min_). Based on these parameters, the joint space asymmetry (JSW.AS) was calculated as the ratio between JSW_max_ and JSW_min_ [21]. The periarticular regions of the radius and the second and third MCP joints were analyzed for the presence, number, maximum size, and anatomical distribution of erosions and osteophytes, using the method described by Stach et al [49]. An example of the joint analysis result is shown in Fig. 2.

#### DXA and VFA

Areal BMD was assessed for the lumbar spine and the hip using the same device (Hologic, Inc., Marlborough, MA, USA), and the T-score for each site was calculated.

Daily quality control procedures, including a standardized phantom, were in place, and the CV was low (1.09% at lumbar spine, 1.15% at total hip, and 1.77% at femoral neck [50]).

Furthermore, vertebral fracture analysis (VFA) of the lumbar and thoracic spine was performed to assess any vertebral fractures using the semiquantitative method described by Genant et al [51]. VFA images were evaluated by 2 of the investigators (C.R. and J.D.), and the results were compared. Any disagreements were settled by consensus. Based on the VFA results, each participant's spinal deformity index (SDI) was calculated as the sum of Genant grades [52].

### Bone impact microindentation

Using an OsteoProbe® (Active Life Scientific, Santa Barbara, CA, USA), bone integrity was assessed at the tissue level via microindentation of the medial non-dominant tibia. With the participant in a supine position, the indentation site was identified as the midpoint between the medial malleolus and the tibial plateau, using a measuring tape. After skin disinfection, an intracutaneous and periosteal local anesthetic was applied. The tip of the OsteoProbe stylus was inserted through the skin perpendicular to the tibia, and a series of indentations was performed, as guided by the hardware supplied by the manufacturer. A minimum of 8 indentations was made, and the stylus tip was moved slightly between each indentation. Finally, a series of indentations was performed on a polymer reference block, after which the participant's bone material strength index (BMSi) was calculated and presented on the system supplied by the manufacturer.

Interobserver variability was investigated through repeated measures in 12 participants, yielding a CV of 10.1%.

### Biochemical measurements

With the participant fasting, blood samples were drawn and stored at −80°C until study termination. Subsequently, the samples were analyzed for parathyroid hormone (PTH), 1,25-Vitamin D, sclerostin, collagen I beta-crosslaps (CTx), and procollagen I N-terminal propeptide (P1NP).

### Questionnaires

Self-reported lower limb joint pain and stiffness were assessed using the Danish version of the Western Ontario and McMaster Universities Osteoarthritis Index (WOMAC) scale [53] including a total of 24 questions across 3 domains (pain, stiffness, and activities of daily living impairment). Scores range from 0 to 96, with higher scores indicating more pain and stiffness. Participants with acromegaly also completed the Danish version of the AcroQoL questionnaire [54], with higher scores reflecting better QoL.

### Statistical analysis

The distribution of continuous data was assessed visually using QQ-plots. Normally distributed continuous data were presented as mean (SD) and non-normally distributed data as median [IQR]. For group comparison, dichotomous data were analyzed using the Chi-square test, normally distributed data using the two-sample *t*-test, and non-normally distributed data using the rank-sum test. Log-normally distributed data were analyzed using the two-sample *t*-test and the obtained estimates back-transformed using exponentiation. Regression analyses for dichotomous, count, and continuous outcomes were performed as binary (log-link), negative binomial, and linear regression, respectively. Linear regression model validity was assessed using Q–Q-plots of the residuals, and the likelihood ratio test for overdispersion was used to assess negative binomial regression model validity. Due to their well-established deleterious effect on bone health [47, 55], regression analyses for bone-related outcomes were adjusted for smoking and diabetes status, whereas those for joint-related outcomes were not. A 5% significance level was assumed. Data analysis was performed using STATA v. 17 for Mac.

## Results

The study included 17 patients with acromegaly (mean age 61.8 ± 11.9 years, 65% female) and 17 matched controls (mean age 61 ± 11.7 years, 65% female). The majority of women were postmenopausal, with only 2 (18%) and 3 (27%) premenopausal women in the acromegaly and control groups, respectively.

No participants with acromegaly had Multiple Endocrine Neoplasia 1-associated acromegaly.

Participants with and without acromegaly were similar as regards body mass index (BMI), menopausal status, diabetes status, and diabetes duration. A significantly larger proportion of controls had never smoked (88.2% vs 35.3%, *P* = .004), whereas the mean number of pack years among smokers was similar (*P* = .18) (Table 1).

**Table 1:** Demographic data for participants and disease-specific data for the acromegaly group

		Acromegaly (*n* = 17)	Controls (*n* = 17)
Age at inclusion (years), mean (SD)		61.8 (11.9)	61.0 (11.7)
Sex, *n* (%) female		11 (65%)	11 (65%)
Menopausal status	Pre-menopausal	2 (18%)	3 (27%)
	Post-menopausal	9 (82%)	8 (73%)
BMI (kg/m^2^), mean (SD)		29.7 (6.6)	27.8 (5.4)
Smoking status	Current daily smoker, *n* (%)	1 (5.9%)	1 (5.9%)
	Previous daily smoker, *n* (%)	10 (58.8%)	1 (5.9%)
	Never smoked, *n* (%)	6 (35.3%)	15 (88.2%)
Pack years (years), mean (SD)		11.9 (5.4)	25.5 (34.6)
Patients with type 2 diabetes, *n* (%)		6 (35.3%)	7 (41.2%)
Diabetes duration (years), mean (SD)		6.5 (5.6)	8.4 (3.4)
Age at acromegaly diagnosis (years), mean (SD)		48.6 (12.7)	—
Acromegaly duration (years), mean (SD)		13.1 (8.8)	—
Surgically treated, *n* (%)		17 (100%)	—
Medical treatment, *n* (%)		9 (53%)	—
Latest IGF-I xULN, mean (SD)		0.9 (0.4)	—
Latest IGF-I SDS, mean (SD)		1.2 (1.6)	—
Patients with hypopituitarism, *n* (%)		4 (24%)	—
AcroQoL score, mean (SD)		80.9 (14.3)	—

Abbreviations: BMI, body mass index; IGF-I SDS, Insulin-Like Growth Factor 1 Standard Deviation Score.; IGF-I xULN, Insulin-Like Growth Factor 1 times upper limit of normal; SD, standard deviation.

All participants with acromegaly were treated surgically, and 9 received adjuvant medical therapy, including somatostatin analogs (*n* = 8), pegvisomant (*n* = 1) and cabergoline (*n* = 1). Biochemical control of acromegaly was achieved in all patients, with a mean IGF-I standard deviation score of 1.2 (±1.6) at the latest outpatient visit. Four participants with acromegaly received hormone replacement therapy on one or more axes, of which one was treated with sex hormone replacement. All other pituitary axes were intact, and the substituted hormones were within normal levels (data not shown). The mean AcroQoL score was 80.9 (±14.3) points (Table 1).

### Joint-related parameters

**Figure 3:**
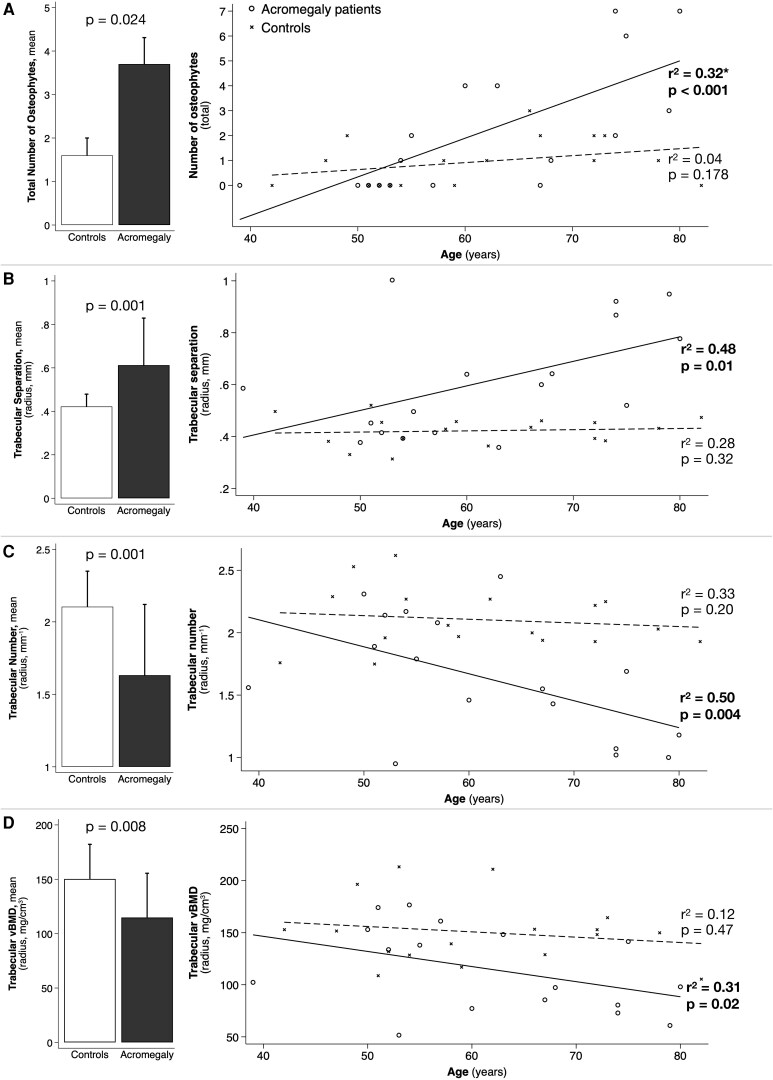
Joint- and bone-related parameters, mean and SD, and plotted against participant age. A) total osteophyte number, B) trabecular separation at radial site, C) trabecular number at radial site, and D) trabecular vBMD at radial site. Circles and solid line: acromegaly group; crosses and dashed line: control group. Abbreviations: TbSp, trabecular separation; TbN, trabecular number; vBMD, volumetric bone mineral density.

Participants with acromegaly had a significantly higher mean JSW_max_ of the MCP joints than controls (MCP2: 2.92 (0.14) mm vs 2.80 (0.15) mm, *P* = .037; MCP3: 2.85 (0.13) mm vs 2.68 (0.17) mm, *P* = .004), and a significantly larger proportion of patients had at least one osteophyte in the third MCP joint (50% vs 11.8%, *P* = .017). Participants with acromegaly tended to have a higher median number of osteophytes at all joint sites (Table 2). In regression analyses, the total number of osteophytes correlated positively with the presence of acromegaly (*β*: .84 [0.11; 1.57], *P* = .024) (Table 3) and with increasing age in the acromegaly group (*β*: .08 [0.04; 0.12], *P* < .001), but not the control group (*β*: .03 [−0.013; 0.07], *P* = .178) (Fig. 3A). Both the number and size of osteophytes at the third MCP joint correlated positively with the presence of acromegaly (*β*: 1.79 [0.27; 3.32], *P* = .021, and *β*: .28 [0.03; 0.52], r^2^ = 0.16, *P* = .034, respectively) (Table 3).

**Table 2:** Joint-related outcomes

HRpQCT joint measurements												
	Radioscaphoid			Radiolunate			MCP2			MCP3		
Variable	Acromegaly	Controls	*P* =	Acromegaly	Controls	*P* =	Acromegaly	Controls	*P* =	Acromegaly	Controls	*P* =
JSV (mm^3^), mean (SD)	224.71 (76.97)	220.52 (66.98)	.87	211.56 (49.50)	192.54 (62.23)	.36	107.37 (29.09)	92.62 (24.21)	.13	114.60 (35.02)	96.78 (26.73)	.12
JSW (mm), mean (SD)	1.99 (0.36)	1.86 (0.28)	.29	2.13 (0.26)	2.11 (0.32)	.89	1.85 (0.32)	1.76 (0.27)	.39	1.78 (0.33)	1.62 (0.26)	.15
JSW_max_ (mm), mean (SD)	2.97 (0.15)	2.88 (0.16)	.13	3.02 (0.13)	3.02 (0.14)	.93	2.92 (0.14)	2.80 (0.15)	.**037**	2.85 (0.13)	2.68 (0.17)	.**004**
JSW_min_ (mm), median [IQR]	1.07 [0.16-1.23]	0.98 [0.16-1.23]	.76	0.16 (0.16-1.07)	0.16 (0.16-0.86)	.85	0.74 (0.16-1.23)	1.02 (0.16-1.19)	.90	0.90 [0.16-1.23]	0.86 [0.16-1.19]	.75
JSW asymmetry, median [IQR]	2.86 (2.40-17.50)	2.82 (2.31-16.50)	.55	17.00 (2.77-18.50)	17.50 (3.34-18.75)	.91	3.89 (2.33-18.00)	2.72 (2.35-17.00)	.63	3.00 (2.27-16.50)	3.17 (2.25-15.50)	.87
Osteophytes (yes/no), *n* (%)	2 (11.8%)	5 (29.4%)	.20	—	—	—	9 (56.3%)	7 (41.2%)	.39	8 (50.0%)	2 (11.8%)	.**017**
Osteophyte number, median* [IQR]	2 [1-2]	1 [1-1]	.11	—	—	—	2 [1-3]	1 [1-2]	.14	1 [1-2]	1 [1-1]	.33
Osteophyte max. diameter (mm), mean (SD)	1.0 (0.1)	1.8 (0.3)	.**01**	—	—	—	1.5 (0.7)	1.0 (0.4)	.087	1.2 (0.3)	0.9 (0.1)	.20

*P* < .05 marked in bold.

Abbreviations: JSV, Joint space volume; JSW, Joint space width; MCP2, second metacarpophalangeal joint; MCP3, thirdmetacarpophalangeal joint.

^*^Among patients with ≥ 1 osteophyte at a given joint.

**Table 3:** Regression analyses of joint- and bone-related outcomes by acromegaly status (patients vs controls)

Joint-related outcomes					
Variable		*β*/RR [95%CI]	*P* =	r^2^	Regression model
Presence of osteophytes, any (yes/no)		RR: 1.00 [0.57; 1.75]	1.00	—	Binary (log-link)
Osteophyte count, total		*β*: .84 [0.11; 1.57]	**.024**	—	Negative binomial
Radius	Ostephyte number	*β*: .41 [−1.03; 0.84]	.579	—	Negative binomial
	Osteophyte size	*β*: −.74 [−1.13; −0.34]	.**004**	0.70	Linear
MCP2	Osteophyte number	*β*: .89 [−0.10; 1.89]	.078	—	Negative binomial
	Osteophyte size	*β*: .46 [−0.10; 1.01]	.099	0.12	Linear
MCP3	Osteophyte number	*β*: 1.79 [0.27; 3.32]	.**021**	—	Negative binomial
	Osteophyte size	*β*: .28 [0.03; 0.52]	.**034**	0.16	Linear

Bone-related outcomes Adjusted for smoking and diabetes status					
Variable		*β*/RR [95%CI]	*P* =	r^2^	Regression model
Presence of vertebral deformities (yes/no)		RR: 1.99 [1.17; 3.38]	**.011**	—	Binary (log-link)
Number of vertebral deformities		*β*: 1.10 [0.35; 1.85]	**.004**	—	Negative binomial
Spinal deformity index		*β*: 1.34 [0.26; 2.42]	**.017**	0.36	Linear
Radius	Trabecular vBMD	*β*: −35.57 [−73.33; 2.19]	.064	0.23	Linear
	Bone volume/tissue volume	*β*: −.03 [−0.06; 0.002]	.063	0.23	Linear
	Trabecular number	*β*: −.51 [−0.87; −0.15]	.**008**	0.30	Linear
	Trabecular thickness	*β*: .001 [−0.01; 0.01]	.814	0.14	Linear
	Trabecular separation	*β*: .21 [0.04; 0.38]	.**016**	0.30	Linear
Tibia	Trabecular vBMD	*β*: −34.27 [−70.11; 1.57]	.06	0.27	Linear
	Bone volume/tissue volume	*β*: −.03 [−0.06; 0.001]	.059	0.27	Linear
	Trabecular number	*β*: −.32 [−0.72; 0.09]	.119	0.13	Linear
	Trabecular thickness	*β*: −.01 [−0.012; 0.001]	.12	0.28	Linear
	Trabecular separation	*β*: .12 [−0.005; 0.25]	.059	0.21	Linear

Abbreviations: 95%CI, 95% confidence interval; BV/TV, bone volume to tissue volume ratio; MCP2, second metacarpophalangeal joint; MCP3, third metacarpophalangeal joint; RR, risk ratio; vBMD, volumetric bone mineral density.

Participants with acromegaly had significantly higher (ie, worse) median WOMAC-scores than controls (17.0 vs 1.0, *P* = .002).

The remaining HR-pQCT-derived joint measurements were similar between the 2 groups (Tables 2 and 3), as were the anatomical distribution and Stach grade of osteophytes and erosions (data not shown).

### Bone-related parameters

A significantly larger proportion of participants with acromegaly had at least one vertebral deformity (Genant grade ≥1), as demonstrated by VFA (15 vs 8, *P* = .01), a higher mean number of deformed vertebrae per participant (2.1 (±1.3) vs 0.7 (±0.8), *P* < .001), and a higher mean SDI (2.6 (±2.0) vs 1.3 (±1.5), *P* = .04) (Table 4). Participants with acromegaly had significantly lower trabecular vBMD at both sites (radius: 114.8 (±40.7) vs 150.3 (±31.9) mg/cm^3^, *P* = .008, tibia: 129.3 (±40.2) vs 166.2 (±22.0) mg/cm^3^, *P* = .002) and BV/TV (radius: 0.10 (±0.03) vs 0.13 (±0.03), *P* = .008, tibia: 0.11 (±0.03) vs 0.14 (±0.02), *P* = .002), as well as a higher trabecular separation (radius: 0.58 (±1.40) vs 0.42 (±1.15) mm, *P* = .001, tibia: 0.50 (±0.15) vs 0.41 (±0.062) mm, *P* = .023). Likewise, the acromegaly group had significantly lower mean trabecular thickness of the tibia (0.056 (±0.01) mm vs 0.064 (±0.01) mm, *P* = .005), and fewer trabeculae per millimeter at the radial site (1.63 (±0.49) vs 2.11 (±0.25) mm^−1^, *P* = .001) (Table 4).

**Table 4:** Bone-related outcomes

DXA, VFA, OsteoProbe, and biomarker measurements			
Variable	Acromegaly	Controls	*P* =
T-score, hip, mean (SD)	−0.4 (1.0)	−0.4 (1.0)	.97
T-score, spine, mean (SD)	0.4 (2.1)	−0.1 (1.3)	.42
Presence of vertebral deformities, *n* (%)	15 (88%)	8 (47%)	.**01**
Number of vertebral deformities, mean (SD)	2.1 (1.3)	0.7 (0.8)	**<**.**001**
Spinal deformity index, mean (SD)	2.6 (2.0)	1.3 (1.5)	.**04**
Bone material strength index, mean (SD)	76.8 (9.2)	82.0 (5.4)	.059
CTX (ng/mL), mean (SD)	0.3 (0.3)	0.3 (0.2)	.77
P1NP (ng/mL), mean (SD)	56.8 (22.4)	64.4 (27.6)	.38
Sclerostin (pmol/L), mean (SD)	116.5 (46.6)	136.8 (36.5)	.17
25-OH-VitD (nmol/L), mean (SD)	101 (31.0)	92.8 (37.0)	.59
1,25-OH-VitD (pg/mL), mean (GSD)	61.8 (×/1.5)	52.3 (×/1.3)	.14
PTH (pmol/L), mean (SD)	5.2 (1.8)	5.1 (1.5)	.81

HRpQCT bone measurements						
	Radius			Tibia		
Variable	Acromegaly	Controls	*P* =	Acromegaly	Controls	*P* =
Cortical area (mm^3^), mean (SD)	71.4 (25.0)	60.2 (16.3)	.13	126.5 (33.3)	123.4 (29.8)	.78
Cortical perimeter (mm), mean (SD)	80.0 (9.4)	78.3 (10.8)	.63	113.5 (10.3)	110.8 (11.9)	.49
Cortical vBMD (mg/cm^3^), mean (SD)	905.7 (58.2)	872.2 (88.4)	.20	880.8 (61.1)	856.0 (64.2)	.26
Cortical porosity (%), mean (SD)	0.03 (0.01)	0.03 (0.02)	.37	0.06 (0.02)	0.08 (0.03)	.**028**
Cortical pore diameter (mm), mean (SD)	0.16 (0.01)	0.15 (0.01)	.11	0.18 (0.03)	0.18 (0.02)	.72
Cortical thickness (mm), mean (SD)	1.02 (0.33)	0.86 (0.19)	.085	1.23 (0.30)	1.22 (0.26)	.97
Trabecular area (mm^3^), mean (SD)	243.6 (54.1)	265.3 (62.7)	.29	686.4 (137.5)	652.8 (151.6)	.50
Trabecular vBMD (mg/cm^3^), mean (SD)	114.8 (40.7)	150.3 (31.9)	.**008**	129.3 (40.2)	166.2 (22.0)	.**002**
Bone vol./tissue vol., mean (SD)	0.10 (0.03)	0.13 (0.03)	.**008**	0.11 (0.03)	0.14 (0.02)	.**002**
Trabecular number (mm^−1^), mean (SD)	1.63 (0.49)	2.11 (0.25)	.**001**	1.92 (0.48)	2.16 (0.29)	.078
Trabecular thickness (mm), mean (SD)	0.06 (0.01)	0.06 (0.01)	.73	0.056 (0.01)	0.064 (0.01)	.**005**
Trabecular separation (mm), mean (SD)	0.58 (1.41)	0.42 (1.149)	.**001**	0.50 (0.15)	0.41 (0.06)	.**023**

*P* < .05 marked in bold.

Abbreviations: CTx, collagen I beta-crosslaps; DXA, dual X-ray absorptiometry; P1NP, procollagen I N-terminal propeptide; vBMD, volumetric bone mineral density.; VFA, vertebral fracture assessment.

There were no statistically significant differences in HR-pQCT estimates between participants with and without vertebral deformities (data not shown).

In regression analyses with adjustment for confounders (smoking and diabetes status), the risk of having at least one vertebral deformity was higher in the acromegaly group (RR: 1.99 [1.17; 3.38], *P* = .011 (unadjusted *P* = .021)), as was the number of vertebral deformities (*β*: 1.10 [0.35; 1.85], *P* = .004) and the SDI (*β*: 1.34 [0.26; 2.42], *r*^2^ = 0.36, *P* = .017) (Table 3). When including only more severe vertebral deformities (Genant grade ≥ 2), no differences were found between groups (data not shown).

After adjustment for confounders, trabecular number and separation of the distal radius correlated negatively (*β*: −.51 [−0.87; −0.15], *r*^2^ = 0.30, *P* = .008) and positively (*β*: .21 [0.04; 0.38], *r*^2^ = 0.30, *P* = .016) with the presence of acromegaly, respectively (Table 3). Trabecular number and vBMD correlated negatively with age in the acromegaly group (*β*: −.03 [−0.05; −0.01], *r*^2^ = 0.50, *P* = .004 and *β*: −1.95 [−3.52; −0.37], *r*^2^ = 0.31, *P* = .02, respectively), while the correlation with trabecular separation was positive (*β*: .01 [0.00; 0.02], *r*^2^ = 0.48, *P* = .01) (Fig. 3B-3D). No such associations reached statistical significance in the control group.

As regards the cortical measurements, participants with acromegaly had significantly lower cortical porosity of the tibia than controls (0.06 (±0.02) % vs 0.08 (±0.03) %, *P* = .028), but there were no other statistically significant differences regarding cortical bone measurements (Table 4).

No statistically significant differences were found regarding mean DXA-derived *T*-scores of the hip (−0.4 (±1.0) in both groups, *P* = .97) or spine (0.4 (±2.1) vs −0.1 (±1.3), *P* = .42), BMSi (76.8 (±9.2) vs 82.0 (±5.4), *P* = .059), bone turnover markers (CTX: 0.3 (±0.3) ng/mL in both groups, *P* = .77, P1NP: 56.8 (±22.4) vs 64.4 (±27.6) ng/mL, *P* = .38, sclerostin: 116.5 (±46.6) vs 136.8 (±36.5) pmol/L, *P* = .17) or markers of calcium and phosphate metabolism (25-hydroxy vitamin D: 101 (±31.0) vs 92.8 (±37.0) nmol/L, *P* = .59, 1,25-hydroxy vitamin D: 61.8 (×/1.5) vs 52.3 (×/1.3) pg/mL, *P* = .14 and PTH: 5.2 (±1.8) vs 5.1 (±1.5) pmol/L, *P* = .81).

In a subgroup analysis within the acromegaly group, comparing medically treated and surgically cured patients, joint-related HR-pQCT parameters exhibited no statistically significant differences (data not shown).

As for the bone-related HR-pQCT parameters, trabecular indices were significantly more deteriorated in medically treated patients. They had lower tibial vBMD (*β*: −48.79 [−85.20; −12.39], *r*^2^ = 0.44, *P* = .01), lower tibial BV/TV (*β*: −.04 [−0.07; −0.01], r^2^ = 0.44, *P* = .01), lower radial and tibial trabecular number (radius: *β*: −.65 [−1.11; −0.20], r^2^ = 0.56, *P* = .01; tibia: *β*: −.51 [−1.00; −0.01], *r*^2^ = 0.45, *P* = .04) and higher trabecular separation (radius: *β*: .29 [−0.09; −0.50], *r*^2^ = 0.54, *P* = .01; tibia: *β*: .17 [0.02; 0.31], *r*^2^ = 0.49, *P* = .03), after adjustment for smoking and diabetes status.

The 2 subgroups did not differ significantly in WOMAC or AcroQoL scores (data not shown).

**Figure 4:**
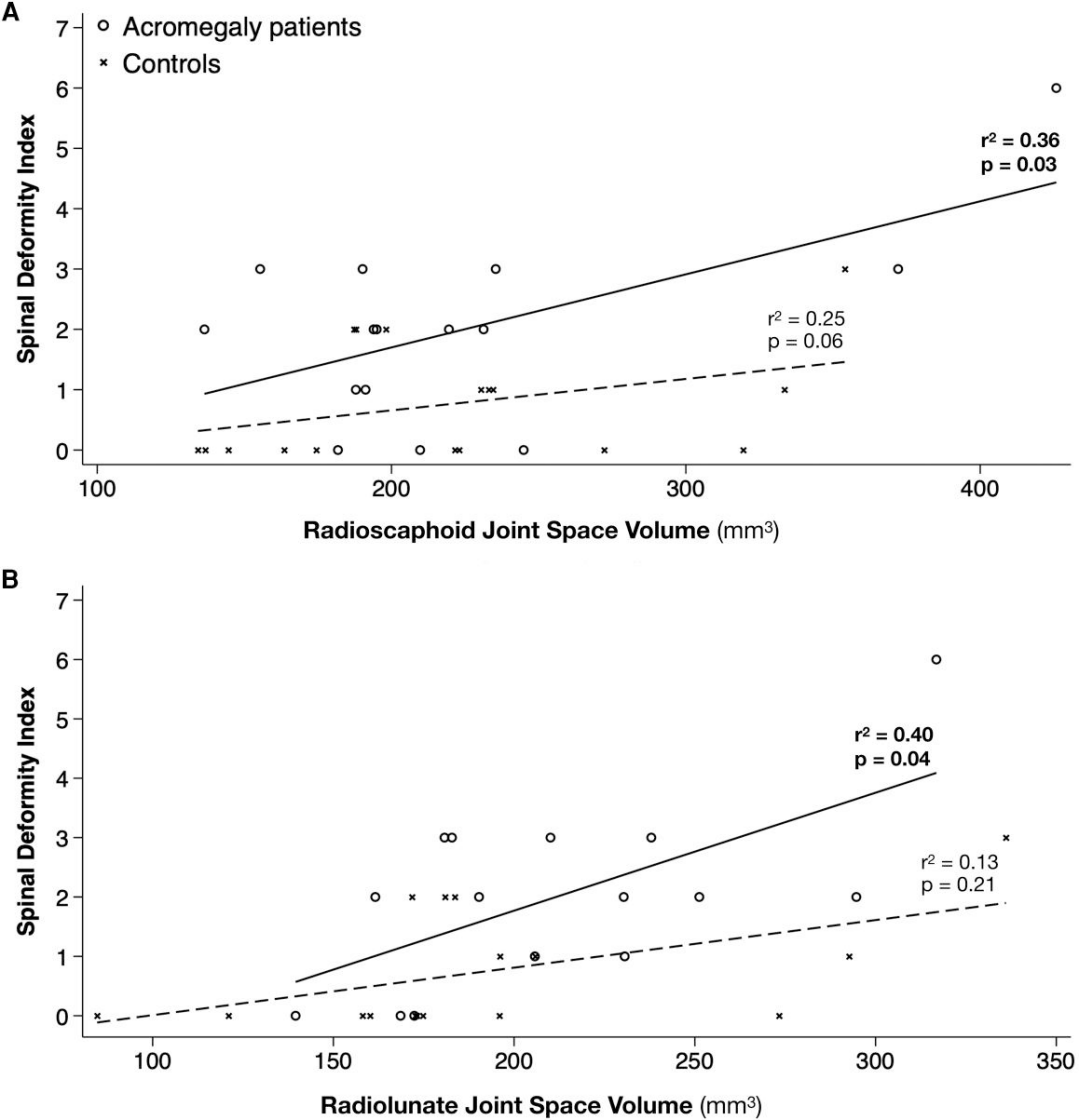
Correlation between spinal deformity index and wrist joint volumes, scatter plot with fitted lines. (A) SDI and radioscaphoid joint volume and (B) SDI and radiolunate joint volume. Circles and solid line: acromegaly group; crosses and dashed line: control group.

When cross-correlating joint- and bone-related parameters, SDI was found to be positively associated with wrist joint volume (radioscaphoid: *β*: .01 [0.00; 0.02], *r*^2^ = 0.36, *P* = .03; radiolunate: *β*: .02 [0.00; 0.04], *r*^2^ = 0.40, *P* = .04) (Fig. 4) in the acromegaly group, while the total number of osteophytes was negatively associated with BMSi (*β*: −.03 [0.06; 0.00], *P* = .05); no such associations were found in the control group. When correlating HR-pQCT-derived joint and bone parameters, a pattern consistently found across all examined joints was an association between joint volume/width measurements (JSV and JSW) and bone size measurements (CtA, CtPm, and TbA); no associations to microstructural bone parameters (TbvBMD, TbTh, TbN, and TbSp) were found (data not shown).

## Discussion

In this study on patients with long-standing, biochemically controlled acromegaly, we have demonstrated a significantly increased burden of joint disease and markedly reduced trabecular bone quality, as defined by HR-pQCT-derived measures. Osteophytosis, particularly in the MCP joints, was highly prevalent in participants with acromegaly, and the maximum joint space width was increased, as was subjective joint pain. Trabecular bone quality was significantly reduced in the acromegaly group, with lower trabecular vBMD, fewer trabeculae, increased trabecular separation, decreased trabecular thickness, and lower bone/tissue volume ratio. Participants with acromegaly also exhibited worse outcomes regarding the presence, number, and severity of vertebral deformities, which were associated with arthropathy markers, ie, wrist joint volume. Together, these findings exemplify and further elucidate the interplay between the skeletal and articular system in the setting of long-term, hormonally controlled acromegaly.

To the best of authors' knowledge, this is the first study examining bone quality using as wide an array of diagnostic modalities, and in patients with a disease duration as long as in the present paper. Moreover, we are the first to present HR-pQCT data on osteophyte number and size measurements in acromegaly. Features unique to HR-pQCT, such as the smaller voxel size, enabled us to study minute articular structures such as osteophytes, as well as accurately quantify joint space characteristics, adding new details on the precise size and number of osteophytes in acromegaly to the existing body of evidence, that mainly reports on the presence and anatomical distribution of osteophytes [12, 13, 15, 18].

Osteophytes were present in 60% of participants with acromegaly, and in larger numbers, compared to controls. This is of clinical relevance, considering that severe osteophytosis correlates with joint pain [13], an important determinant of reduced quality of life in patients with acromegaly [7, 8]. The prevalence of osteophytosis in the present study aligns with what has been found in previous works, where clinical and radiological osteoarthritis of the hand joints has been shown to affect 41-89% of patients with acromegaly, even after long-term remission [12, 13, 18]. In participants with acromegaly, increasing age was positively associated with the total number of hand joint osteophytes; similarly, advanced age has been identified as a risk factor for progression of hand osteoarthritis in acromegaly, also regarding osteophytosis [12]; a pattern also present in patients with rheumatoid arthritis [49]. The fact that age was significantly associated with the number of osteophytes in the acromegaly group, but not the control group, supports the notion of an accelerating or compounding effect of (previous) GH excess on periarticular bone that remains even after normalization of GH levels, as previously hypothesized [12, 17, 56].

In addition to osteophytosis of the MCP joints, we found significantly larger values of JSW_max_ in the acromegaly group, with a tendency toward larger joint space volumes and widths across all examined joints. Accurate quantification of joint space characteristics using HR-pQCT has not previously been reported, but our results are in line with the description of classic acromegalic arthropathy as shown in previous studies using conventional radiographs [12, 16-19] and magnetic resonance imaging [15]. Longitudinal studies [12, 13] have demonstrated significant radiological joint deterioration, particularly of the hands, even years after disease remission. This aligns well with our results and underscores the pervasive nature of acromegalic arthropathy, affecting joints both large and small, weight-bearing and non-weight-bearing, as we have previously demonstrated in an epidemiological setting [10].

Participants with acromegaly had a significantly increased risk of having at least one vertebral deformity, and acromegaly correlated significantly with the number and severity of vertebral deformities, as reflected by the SDI. This was mainly driven by Genant grade 1 vertebral deformities, since no differences were found when considering vertebral deformities with Genant grades ≥ 2. Although milder, grade 1 vertebral deformities may still have significance, given that previous studies have shown a propensity for these to progress, and pre-existing vertebral deformities are a risk factor for developing incident deformities [34, 57]. Moreover, accumulated mild vertebral fractures may cause increased thoracic kyphosis, and the disability it entails, including back pain, impaired sagittal balance, and accelerated degeneration of spinal joints [58].

In keeping with the increased risk of vertebral deformities, we found significantly deteriorated HR-pQCT indices of trabecular bone quality in both the tibia and the radius, compared to controls. Notably, our trabecular vBMD and thickness measurements are markedly lower than previously reported (radius: 133-163 mg HA/cm^3^ and 0.07-0.071 mm; tibia: 121-175 mg HA/cm^3^ and 0.07-0.074 mm, respectively) [23, 31, 32, 59]. Our findings on trabecular number and separation are consistent with previous studies; specifically, the trabecular number falls at the lower end of the reported range (radius: 1.6-1.93 mm^−1^), while trabecular separation is comparatively higher (radius: 0.43-0.52 mm) [23, 31, 32]. These deviations may be due to differences in study populations, as previous studies included younger patients (mean 40-52 years) with shorter disease duration (mean 5.9-9.6 years) [59] and patients with active disease [23, 31, 32], while one study included males only [31]. In contrast, our patient population was comprised of a relatively large proportion of older, postmenopausal women; hence, advanced age and physiological hypogonadism, exacerbated by the effects of GH, may account for the comparatively lower trabecular bone quality we found. Compared to surgically cured acromegaly patients, medically treated patients exhibited worse trabecular bone outcomes, likely reflecting more severe acromegaly and thus higher GH exposure in the latter group, necessitating adjuvant medical treatment; a finding consistent with a previous study on acromegaly patients receiving long-term treatment with pegvisomant [60]. Persistent GH secretion abnormalities despite medical treatment [61] may also play a role, and a direct effect of somatostatin analogs on bone formation [62] cannot be ruled out.

The reduced trabecular bone quality in participants with acromegaly was present even in the setting of normal T-scores, underscoring the reduced utility of DXA scans for assessing bone quality in acromegaly patients [23, 31, 32]. The acromegaly patients in the present study had long-standing disease, mean duration of 13.1 years, and were well-controlled, as reflected by both the normal mean IGF-I SD score and high mean AcroQoL score. Thus, our findings indicate permanent damage to the trabecular bone structure, regardless of disease control, as previously reported [63]; this is underscored by the fact that bone turnover markers did not differ between patients and controls, indicating no active bone remodeling. Furthermore, vitamin D and PTH levels did not differ between groups, suggesting intact calcium homeostasis in both groups. There was, however, a tendency toward lower sclerostin levels in the acromegaly group, which supports the previously suggested hypothesis of compensatory reduction of sclerostin secretion in patients with acromegaly, to counteract the negative effects of GH and IGF-I on bone quality [39]—even in well-controlled patients.

We were unable to demonstrate an association between HR-pQCT indices and vertebral fractures, likely due to the limited sample size; however, a previous, larger study was also unable to detect differences in HR-pQCT parameters between patients with and without vertebral fractures [23]. Conversely, deterioration of several markers of trabecular bone quality (TbN, TbvBMD, and TbSp of the radius) significantly correlated with age in the acromegaly group, but not the control group, suggesting that GH excess accelerates features of the physiological aging of the osteoarticular system.

We found significantly lower cortical porosity at the tibial site in participants with acromegaly, a finding also reported previously [64], whereas others have found no difference at the tibial site, but a significantly higher porosity at the radial site [31]. Differences in mechanical load may explain this disparity, as the distal tibia is subjected to mechanical stress to a higher degree, and thus receives more stimuli for bone remodeling [65], however, this would also be the case for the healthy controls.

Spinal deformity index (SDI) was positively associated with increasing joint space volumes, and total osteophyte number was negatively associated with BMSi in the acromegaly group only. These findings confirm the link between bone and joint disease in acromegaly and illustrate the commonality of the effects of GH excess on multiple osteoarticular structures, both trabecular, cortical, and cartilaginous. This supports the notion [66, 67] that bone and joint disease in acromegaly should be considered 2 interconnected parts of the disease complex that is acromegalic osteoarthropathy.

We acknowledge certain limitations to the present study: the cross-sectional design precluded the determination of any causal relationships, and the study lacked statistical power in certain outcomes. Moreover, the small sample size with a high number of endpoints introduces the risk of type I errors, ie, false positive findings, which should be considered when interpreting our results. Furthermore, the study cohort was somewhat heterogenous in some respects, but key factors such as disease duration and hormonal levels were similar across the study sample, and no patients with active disease were included. Finally, an estimate of active acromegaly duration prior to diagnosis and biochemical remission would have strengthened our results, considering the substantial impact of diagnostic delay on bone health in acromegaly [68]. The strengths of the study include a well-matched control group and the use of several modalities for assessment of osteoarticular quality, thoroughly examining both the cortical and trabecular bone compartments, as well as several measures of joint structure.

In conclusion, joint and bone health is significantly impaired in acromegaly, with osteophytosis and reduced trabecular bone quality as the major components. The correlations between joint- and bone-related parameters support the notion that acromegalic osteoarthropathy can be viewed as an interwoven complex. Our findings indirectly emphasize the importance of early diagnosis and treatment of acromegaly to prevent or alleviate complications.

## Data Availability

The data from the present study are available from the corresponding author upon reasonable request.
